# DNA methylation and prediction of biological age

**DOI:** 10.3389/fmolb.2025.1734464

**Published:** 2026-01-12

**Authors:** Yanfang Chen, Xiangshu Cheng, Shaoping Ji

**Affiliations:** 1 Center for Molecular Medicine, Zhengzhou Health College, Zhengzhou, Henan, China; 2 Translational Medicine Center, Huaihe Hospital Affiliated to Henan University, Kaifeng, Henan, China; 3 Department of Gastroenterology, Huaihe Hospital Affiliated to Henan University, Kaifeng, Henan, China

**Keywords:** aging, biological age, chronic diseases, DNA methylation, epigenetic clock

## Abstract

DNA methylation plays a critical role in gene expression regulation and has emerged as a robust biomarker of biological age. This modification will become heavier or site drift along with aging. Recently, it is termed epigenetic clocks—such as Horvath, Hannum, PhenoAge, and GrimAge—leverage specific methylation patterns to accurately predict age-related decline, disease risk, and mortality. These tools are now widely applied across diverse tissues, populations, and disease contexts. Beyond age-related loss of methylation control, accelerated DNA methylation age has been linked to environmental exposures, lifestyle factors, and chronic diseases, further reinforcing its value as a dynamic and clinically relevant marker of biological aging. DNA methylation is reshaping our understanding of aging and disease risk, with promising implications for preventive medicine and interventions aimed at promoting healthy longevity. However, it must be admitted that some challenges remain, including limited generalizability across populations, an unclear mechanism, and inconsistent longitudinal performance. In this review, we examine the biological foundations of DNA methylation, major advances in epigenetic clock development, and their expanding applications in aging research, disease prediction and health monitoring.

## Introduction

Aging is a complex, multifactorial process that affects nearly all biological systems. While chronological age simply measures the passage of time from birth, biological age reflects the functional state and health of an individual’s tissues and organs (Kiselev et al., 2025). This distinction is critical, as individuals of the same chronological age often exhibit markedly different biological conditions, disease risks, and mortality trajectories (Dugue et al., 2018). Therefore, biological age potentially serves as a more meaningful measure of aging-related decline and is increasingly used to assess overall health status, predict disease onset, and evaluate the effectiveness of interventions aimed at promoting healthy longevity (Dugue et al., 2018; Petkovich et al., 2017).

Among various biomarkers proposed to estimate biological age, epigenetic modifications—particularly DNA methylation—have emerged as one of the most reliable and informative (Dugue et al., 2018). In epigenetics, DNA methylation involves the addition of a methyl group to the 5′ position of cytosine residues, typically at CpG dinucleotides, which can regulate gene expression without altering the underlying DNA sequence. Moreover, DNA methylation can be accurately measured by sequencing at methylated sites with bisulfate treatment (Zhang et al., 2012). Age-related changes in DNA methylation pattern are not random; they occur at specific genomic locations. These methylated sites are picked and constitute come patterns, by which scientists can construct “epigenetic clocks” to precisely estimate a person’s biological age based on their DNA modification. As people grow older, their methylation profiles shift in predictable ways (Kiselev et al., 2025; Horvath, 2013; Horvath and Raj, 2018).

DNA methylation is a particularly promising biomarker for several reasons. First, it is stable and quantifiable across various tissues. Measurement methods included bisulfate treatment, microarray, MeDIP(Methylated DNA immunoprecipitant), methylation-specific PCR, third-generation sequencing and mass spectrometry. All these make it suitable for both cross-sectional and longitudinal studies (Pospiech et al., 2023). Second, methylation data can be obtained using standardized, high-throughput methods, such as next-generation sequencing and microarrays (Barros-Silva et al., 2018). Third, the strong correlation between methylation-based age estimates and various health outcomes—including physically functional decline, cognitive impairment, and all-cause mortality—demonstrates its clinical relevance (Chen et al., 2016). Importantly, deviations between DNA methylation age and chronological age, often termed “epigenetic age acceleration,” have been linked to adverse health effects and may reflect cumulative exposure to environmental stressors (such as Air pollution (Jiang et al., 2025) and radiation (Antwih et al., 2013)) and lifestyle factors (Ryan et al., 2020; Swiatowy et al., 2021) (Figure 2). Interestingly, these changes of DNA methylation majorly occur in the muscles and adipose tissue. However, tissue-specific methylation may increase the difficulty to predict biological aging by DNA methylation.

The mechanism underlying changes in DNA methylation patterns over time remains poorly understood. During aging, DNA methylation patterns undergo dynamic changes, leading to both global hypomethylation and site-specific hypermethylation (Figure 2). These alterations are influenced by a combination of intrinsic and extrinsic factors, including oxidative stress (Goncalves et al., 2021), inflammation (Yan et al., 2023), cellular replication (Vandiver et al., 2015), and environmental exposures (Ryan et al., 2020). At the molecular level, the activity of DNA methyltransferases (DNMTs), particularly DNMT1, DNMT3A, and DNMT3B, declines or becomes dysregulated with age, resulting in imperfect maintenance and *de novo* methylation (Cui and Xu, 2018; Berletch et al., 2007). In parallel, ten-eleven translocation (TET) enzymes involved in demethylation may also be affected (Kolodziej-Wojnar et al., 2023). These changes impact regulatory regions of the genes, such as promoters and enhancers, thereby altering gene expression. Age-related methylation pattern shift or drift contributes to transcriptional noise, genomic instability, and the dysregulation of key pathways associated with aging and disease (Jones et al., 2015; Issa, 2014). Recent research has also led to the development of epigenetic clocks, which track age-related methylation changes at specific CpG sites to estimate biological age, highlighting the predictive power of these molecular shifts (Wang H. T. et al., 2024).

Several well-studied epigenetic clocks, including the Horvath and Raj (2018), Hannum et al. (2013), PhenoAge (Levine et al., 2018), and GrimAge (Lu et al., 2019) models and others, have each made significant contributions to aging research. In addition to DNA methylation, IgG glycosylation patterns are used to estimated biological age as well (Gudelj et al., 2018). Measuring IgG glycan profiles provides insights into immune aging and chronic inflammation, which are key aging hallmarks. The method is less invasive and reflects immune system health but is relatively new, with limited standardization and clinical validation. In addition, lymphocyte grouping examines immune cell subset proportions, revealing immune senescence and systemic aging (Lin et al., 2016). It’s informative for immune status and disease risk but can be influenced by transient infections or conditions, reducing specificity for aging alone. Other methods include telomere length (Vaiserman and Krasnienkov, 2020), which is easy to measure but less predictive of overall aging, and clinical biomarkers reflecting current health rather than cumulative aging. Additional method include Transcriptomic Clocks (Peters et al., 2015), Proteomic Clocks and Metabolomic Clocks (Lehallier et al., 2019; Zhang et al., 2024). However, each method offers different insights and has varying accuracy, cost, and practicality for patients.

In addition, there are other epigenetic clocks: DunedinPACE, a pace-of-aging epigenetic clock that estimates how quickly someone is biologically aging at the time of measurement, rather than their cumulative biological age (Sant’Anna Barbosa Ferreira et al., 2025); DunedinPoAm, it measures the rate of biological aging based on methylation patterns tied to longitudinal physiological changes (Hillary et al., 2020); CausAge, a new methylation model designed to estimate biological age by focusing on methylation changes that are causally linked to aging processes rather than merely correlated with age (Ying et al., 2024). Recently, ELOVL2 clock has been developed. It is a DNA methylation–based age predictor that uses the methylation level of CpG sites in the ELOVL2 gene, as a biomarker because these sites show one of the strongest correlations with chronological age. Collins et al. developed 10-CpG clock, it is another DNA methylation–based epigenetic age predictor, which estimates biological age via using the methylation levels at ten specific CpG sites selected for their strong age-related changes. This simplified model applies a linear combination of methylation at these 10 CpGs (often measured in saliva or other tissues) to predict an individual’s age (James et al., 2025). CheekAge is a next-generation DNA methylation–based epigenetic aging clock trained on cheek (buccal) cell DNA methylation patterns that predicts biological age and is also associated with lifestyle, health factors, and mortality risk. Unlike many traditional clocks developed in blood (Shokhirev et al., 2024). Compared with the first 4 DAN epigenetic clocks, these are recently developed and have not yet widely used.

By synthesizing recent advances and ongoing challenges, this review aims to clarify the utility of DNA methylation as a biomarker of biological age and to underscore its potential in advancing our understanding of aging and improving human health across the lifespan by preventing disease onset.

## Fundamentals of DNA methylation and aging

DNA methylation is a key epigenetic modification involving the addition of a methyl group to the 5-carbon position of cytosine residues, predominantly at CpG dinucleotides. This biochemical process is catalyzed by DNA methyltransferases (DNMTs) and plays a crucial role in regulating gene expression, maintaining genomic stability, and influencing cellular identity (Patrizi et al., 2025). Unlike genetic mutations, DNA methylation does not alter the underlying DNA sequence but modulates the accessibility of DNA to transcriptional machinery. Mechanistically, methylated sites are often organized in clustered patterns known as CpG islands, particularly within promoter regions, where they can significantly influence gene activity (Huang et al., 2025).

As organisms age, DNA methylation patterns undergo dynamic changes that reflect both the natural aging process and cumulative environmental influence (Figure 2). Chronic inflammation and oxidative stress alter DNA methylation by modulating DNMT and TET enzyme activity. These changes occur at two levels: global and site-specific. Globally, aging is often associated with a general decline in DNA methylation, known as global hypomethylation, which can lead to genomic instability and increased mutation rates (Jones et al., 2015). Pro-inflammatory signaling and reactive oxygen species promote site-specific hyper- or hypomethylation, reshaping gene expression programs linked to cellular senescence and functional decline. Furthermore, certain genomic regions, especially CpG islands within promoter regions, tend to experience hypermethylation with age. This site-specific hypermethylation can result in the repression of genes involved in critical cellular processes, such as tumor suppression and DNA repair (Li et al., 2025).

**FIGURE 1:**
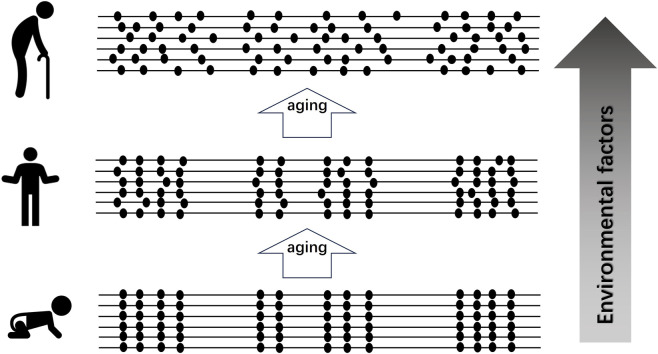
Loss of DNA Methylation Control with Aging and Environmental Factors. DNA methylation is tightly regulated during childhood but gradually becomes less controlled with aging, showing a noticeable decline in older adults (left panel). Environmental factors—such as lifestyle, diet, stress, and exposure to toxins—can further accelerate this loss of regulation. These changes may alter gene expression patterns and contribute to the development of chronic diseases. (Dark dots represent methylated sites on the DNA.).

The age-related remodeling of DNA methylation landscapes forms the basis of “epigenetic clocks” that estimate biological age by quantifying methylation at specific CpG sites (Zheng et al., 2024; Lee et al., 2019). These clocks harness the predictability of methylation changes, serving as sensitive indicators of cellular aging and functional decline beyond chronological time (Figure 1).

Mechanistically, DNA methylation is intertwined with several aging processes. Altered methylation patterns can disrupt gene expression networks, leading to impaired cellular functions such as reduced regenerative capacity, increased inflammation, and metabolic dysregulation (Gudelj et al., 2018). For instance, it has been well known that hypermethylation-induced silencing of anti-oncogenes or DNA repair genes may accelerate the accumulation of genomic damage, even leading to tumorigenesis. Additionally, epigenetic changes can modulate stem cell exhaustion and senescence, further contributing to tissue aging (Krepelova and Neri, 2023). Environmental factors such as poor lifestyle, unhealthy diet, stress, and exposure to toxins also influence methylation patterns, suggesting DNA methylation acts as an integrative biomarker reflecting both intrinsic aging and extrinsic factors (Alfimova et al., 2022).

Pattern change of DNA methylation represents a fundamental biological mechanism tightly linked to aging, capturing the complex interplay between genetic regulation, environmental influences, and cellular function (Figure 1). Understanding these dynamics provides critical insights into the molecular underpinnings of aging and highlights potential avenues for interventions aimed at reducing risk of chronic diseases and promoting healthy longevity.

## Development of epigenetic clocks

The quantification of biological age using DNA methylation has evolved rapidly over the past years, transforming our understanding of aging biomarkers. Early efforts focused on identifying specific CpG sites whose methylation levels correlated strongly with chronological age (Jain et al., 2024; Naue et al., 2017; Fan et al., 2021). However, a study has demonstrated that methylation changes at specific genomic loci, selected in a cohort of 2,840 patients with chronic diseases, can serve as molecular markers for estimating biological age more accurately than chronological age alone (Wang J. et al., 2024).

A major breakthrough came with the development of the first widely recognized epigenetic clocks. The Horvath clock is a pioneering multi-tissue predictor based on 353 CpG sites selected through elastic net regression (Horvath, 2013). Its design enabled accurate age estimation across diverse tissues, making it a versatile tool for aging calculation. Shortly thereafter, the Hannum clock, derived primarily from blood samples, provided another robust age predictor using a distinct set of CpG sites (Hannum et al., 2013). Both clocks showed strong correlations with chronological age and established the feasibility of methylation-based age estimation.

Building on these foundations, newer clocks were developed to improve predictive power for health outcomes beyond chronological aging. The PhenoAge clock combines blood tests and DNA methylation data and integrates clinical biomarkers with methylation data (including 513 CpG sites) to estimate a biological age associated with physiological decline and morbidity risk (Levine et al., 2018). Similarly, the GrimAge clock incorporates DNA methylation surrogates for plasma proteins (including plasminogen activator inhibitor 1 and growth differentiation factor) and smoking history, enhancing its ability to predict lifespan and disease risk more accurately than earlier models (Lu et al., 2019).

Methodologically, the construction of these clocks relies on advanced statistical approaches like penalized regression, machine learning algorithms, and cross-validation to select CpG sites that best capture age-related methylation dynamics (He et al., 2021).It is possible to prioritize CpG sites strongly associated with chronological age but weakly correlated with disease, cell composition, or technical confounders, meanwhile penalize sites showing instability or non-age-related variability in the age prediction model. Improvements in high-throughput methylation profiling technologies have also facilitated the generation of large, diverse datasets critical for clock training and validation (Shokhirev and Johnson, 2025). Different datasets should be used to develop epigenetic clocks for biological age prediction, in which challenges in training and validation across populations will be highlighted, particularly non-European groups, due to genetic, environmental, and methylation differences. These efforts will be benefit to produce generalizability in biological age estimation.

**FIGURE 2:**
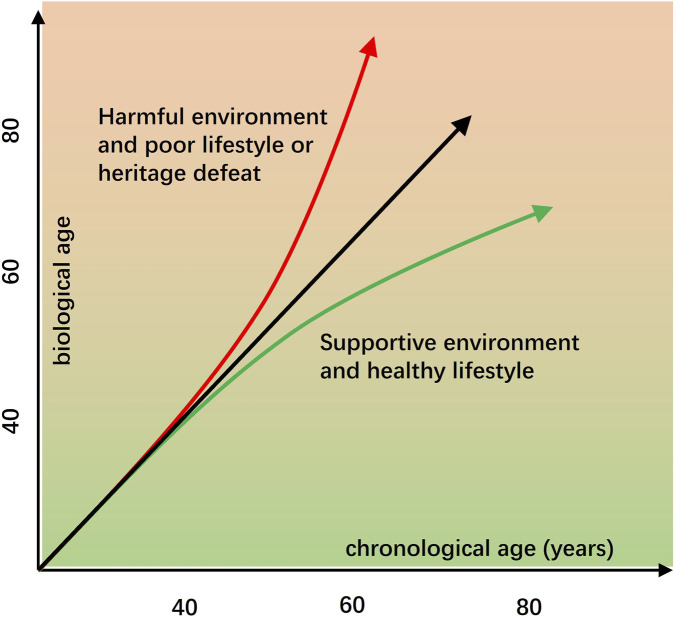
Biological age(clock) usually is different from chronological age. Biological age, estimated through epigenetic clocks based on DNA methylation patterns, often differs from chronological age. Unlike chronological age, which reflects time passed, but biological age indicates the body functional and cellular aging status. Aging can be influenced by both endogenous factors (such as genetics and internal metabolic processes) and exogenous factors such as environmental exposures, diet, stress, pollution, and lifestyle choices. Moreover, that poor lifestyle habits and toxic environments can accelerate biological aging, while regular exercise, a nutrient-rich diet, stress reduction, and clean environments may slow or even partially reverse epigenetic aging, promoting healthier aging outcomes.

**FIGURE 3:**
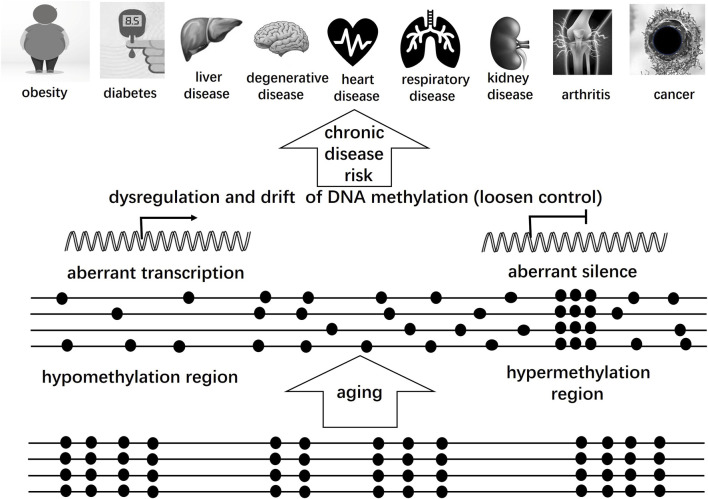
Dysregulation and drift of DNA methylation with aging. Tight control of DNA methylation pattern gradually becomes loosen with aging, including Endogenous and Exogenous factors. A common phenomenon is the pattern drift of DNA methylation and dysregulation of methylation processing, leading to aberrant hypomethylation and aberrant hypermethylation. As a result, gene(s) in hypomethylation methylation region will express, such as oncogenes or other harmful genes with overexpression. In contrast, expression of gene(s) in hypermethylation region will be suppressed, such as anti-oncogenes and immune-related genes. These change of DNA methylation pattern play a crucial role in development of chronic diseases.

These epigenetic clocks exhibit impressive accuracy, with correlations to chronological age often exceeding 0.90 in validation cohorts. Beyond age prediction, they have been applied to assess biological aging in various contexts, including risk of chronic diseases, disease progression, lifestyle interventions, environmental exposures, and even pharmacological treatments (Figure 3). These measures can reflect “epigenetic age acceleration,” which represents deviations from chronological age. Conversely, beneficial environmental factors can slow biological aging, promoting a healthier lifespan (Figure 2). These studies provide valuable insights into individual aging trajectories and associated health risks.

The development of epigenetic clocks represents a significant advancement in aging biology, providing precise and clinically relevant biomarkers of biological age. These tools can guide lifestyle consultations, aid in disease prevention, and improve therapeutic strategies. Ongoing refinement of these models promises to enhance their applications in personalized medicine, epidemiology, and aging intervention studies.

## Applications of DNA methylation clocks in health and disease

DNA methylation clocks have rapidly become invaluable tools for understanding the biological aging process and its relationship to health outcomes. One of the most compelling findings is the strong correlation between methylation-based biological age estimates and various clinical endpoints, including morbidity and mortality. Studies consistently demonstrate that individuals whose DNA methylation age exceeds their chronological age—a phenomenon known as epigenetic age acceleration, facing higher risks of all-cause mortality, frailty, cognitive decline, and functional impairment (Fransquet et al., 2019). This predictive capacity positions methylation clocks as sensitive biomarkers for assessing overall health and longevity beyond traditional risk factors (Figure 3).

However, DNA methylation age is only strongly associated with many chronic diseases. The current evidence suggests it is largely a correlative biomarker rather than a proven direct driver of disease, not a contribution to the pathogenesis of these chronic diseases. Many studies shown that an accelerated methylation age likely reflects cumulative exposure to pathogenic processes (e.g., inflammation, oxidative stress), although specific methylation changes at functional loci may contribute causally in certain contexts. Therefore, it is possible that further investigation will provide an accurate disease prediction in future.

Lifestyle and environmental factors significantly influence DNA methylation age acceleration. For example, smoking, poor diet, physical inactivity, and chronic psychological stress have all been linked to faster epigenetic aging (Quach et al., 2017). Conversely, healthier behaviors, such as regular exercise and balanced nutrition, are associated with slower biological aging (Figure 2). Environmental exposures to pollutants and toxins similarly accelerate methylation aging, highlighting how external factors become biologically embedded via epigenetic modifications (Song et al., 2022). These explorations emphasize the dynamic nature of DNA methylation as a biomarker that integrates genetic, environmental, and lifestyle influences on aging.

Methylation clocks have also been applied extensively in the context of age-related diseases (Figure 3). Elevated epigenetic age acceleration has been observed in individuals with cardiovascular disease, type 2 diabetes (Miao et al., 2024), neurodegenerative disorders such as Alzheimer’s disease (Eissman et al., 2025), and various cancers (Yu et al., 2020; Johnstone et al., 2022). In many cases, methylation age predicts disease onset and progression better than chronological age. Clinically, this has opened new avenues for risk stratification, early examination and diagnosis will benefit patients with a better prognostic outcoming than traditional physical examination, although DNA methylation clocks are not yet routinely used in clinical practice. Clinic practicers estimate biological age from blood or tissue DNA methylation profiles, helping assess aging rate, disease risk, or treatment effects in cohorts. For instance, tracking methylation age in patients undergoing interventions may help evaluate the efficacy of lifestyle changes or pharmacological treatments (such as metformin) aimed at slowing biological aging (Li et al., 2022).

Moreover, DNA methylation clocks hold promise in personalized medicine, enabling personized prevention and specific treatment strategies based on an individual’s biological age and disease prediction, rather than common chronological age. Current research is exploring this integration into routine clinical assessments and population health studies to improve health and lifespan, reducing age-related disease burden. Therefore, it is expected that DNA methylation clocks can provide a robust framework for linking biological aging with health profile, offering transformative potential for understanding, preventing, and managing age-associated diseases. Final challenge is that limited clinical validation, population-specific bias, unclear causality, high cost, tissue specificity, and lack of standardized protocols still limit clinical adoption.

## Current challenges and limitations

Despite the significant advances and promising applications of DNA methylation clocks, several challenges and limitations remain that must be addressed to fully harness their potential.

One major issue is the generalizability of epigenetic clocks across different tissues, populations, and ethnicities (Watkins et al., 2023). Many widely used clocks, such as Horvath’s pan-tissue clock, were developed using predominantly European ancestry cohorts and may not perform equally well in diverse populations (Horvath et al., 2016). Ethnic and genetic variability (on-technical level) can influence baseline methylation patterns, potentially biasing age estimates and reducing accuracy in underrepresented groups. Similarly, tissue-specific methylation signatures mean that clocks trained on blood samples may not reliably reflect biological aging in other organs such as the brain, liver, or muscle (Lokk et al., 2014). In addition, using saliva as sample, instead of blood or tissue for DNA methylation–based biological age prediction will potentially reduce costs while maintaining reasonable accuracy. Other advantages are less invasive and more convenient compared to blood or tissue samples. These differences may be due to the inherent inconsistency in the levels of gene methylation among different organs of the human body.

Another challenge lies in the biological interpretation of epigenetic age acceleration. While accelerated methylation age is consistently associated with adverse health outcomes, the causal mechanisms underlying these associations remain unclear, including detail methylated spots in chrisoms, cells or organs (Simms, 2025). It is still debated whether epigenetic age acceleration actively contributes to aging and disease or merely serves as a biomarker reflecting other underlying processes. Furthermore, different clocks may capture distinct aspects of aging biology, complicating comparisons across studies and raising questions about what exactly “biological age” represents at the molecular level (Yang et al., 2023). Disentangling these mechanisms is essential for translating epigenetic clocks into effective interventions.

Technical and reproducibility concerns also limit the widespread clinical adoption of methylation-based age estimators. As we know, clinical diagnosis needs accurate value or positive or negative markers, but DNA methylation cannot provide accurate value for determination. Moreover, it’s measurement technologies, such as human error in microarrays and bisulfite sequencing, can vary in sensitivity, coverage, and data quality. Batch effects, sample processing differences, and bioinformatic pipelines contribute to variability, making cross-study comparisons challenging. Additionally, longitudinal stability and repeatability of methylation age estimates require further validation to ensure reliability in monitoring aging trajectories or treatment responses (Mei et al., 2023). Standardization of laboratory protocols and analytical methods is crucial for improving reproducibility and clinical utility.

Therefore, while DNA methylation clocks are powerful tools for studying biological aging, addressing issues of population diversity, mechanistic understanding, and technical robustness is vital. Overcoming these challenges will enhance the accuracy, interpretability, and translational impact of epigenetic age estimators in aging research and medicine. Further in-depth research will improve the accuracy of biological age prediction and its association with chronic diseases, while the integration of mega data will help advance this epigenetic clock development. It is believable that measurement of biological age will facilitate human disease prevention and health in future.

At last, Ethical concerns arise when DNA methylation–based biological age is used beyond research, especially if applied to insurance underwriting, employment screening, or medical decision-making. Such use may lead to discrimination, breaches of genetic privacy, or overinterpretation of probabilistic risk. Clear regulatory frameworks, data protection, informed consent, and clinical context are essential to prevent abuse and ensure fair application.

## Future directions and emerging trends

The field of DNA methylation and biological age is rapidly evolving, with exciting new directions poised to enhance our understanding of aging and improve clinical applications. Among these, multi-omics integration, personalized aging measures, targeted interventions, and ethical considerations stand out as pivotal areas shaping the future landscape. To integrate multi-omics by combining DNA methylation with such as transcriptomics, proteomics, and metabolomics in machine-learning models will capture ageing biology completely. For example, OMICmAge uses methylation plus proteomic and metabolomic data to improve prediction and disease associations over methylation alone (Chen et al., 2023). Large multi-omics aging clocks uncover distinct ageing subtypes and pathways, showing better precision and biological insight than single-omic clocks.

As individuals age, the precise regulation of DNA methylation gradually deteriorates, leading to widespread epigenetic drift. This loss of control results in both global hypomethylation and site-specific hypermethylation, disrupting normal gene expression patterns. Global hypomethylation can lead to genomic instability, activation of transposable elements, and oncogene expression, while localized hypermethylation may silence tumor suppressor genes or genes critical for immune regulation and metabolic function. These changes are increasingly recognized as contributors to the development of chronic diseases. For example, aberrant DNA methylation patterns have been implicated in cancer, cardiovascular disease, type 2 diabetes, and neurodegenerative disorders such as Alzheimer’s disease (Wang J. et al., 2024). Inflammation and oxidative stress—common in aging—further accelerate methylation abnormalities, creating a feedback loop that promotes disease progression (Wang et al., 2025). Moreover, environmental exposures, poor lifestyle habits, and chronic stress can exacerbate methylation dysregulation, even early in life, compounding long-term disease risk. As a result, DNA methylation is not only a marker of biological aging but also a mechanistic link between aging and chronic disease development.

One of the most promising trends is the integration of DNA methylation data with other layers of biological information, such as transcriptomics, proteomics, metabolomics, and microbiomics. This multi-omics approach offers a holistic view of aging by capturing complex molecular interactions/network that DNA methylation alone cannot fully explain. Combining these datasets can refine biological age estimates, identify novel aging biomarkers, and uncover mechanisms driving age-related functional decline. Advances in machine learning and systems biology enable the development of personalized aging profiles that incorporate individual variability (non-tech level) in genetics, epigenetics, and environmental exposures (Chen et al., 2023). Such comprehensive metrics promise to revolutionize personalized medicine by tailoring prevention and treatment strategies based on an individual’s unique aging trajectory.

Parallel to these analytical advances, there is growing interest in interventions targeting epigenetic aging (Li et al., 2024). Lifestyle modifications, including diet, exercise, and stress management, have demonstrated potential to modulate DNA methylation patterns and slow epigenetic age acceleration. Pharmacological approaches, such as senolytics, epigenetic modulators, and novel small molecules, are under investigation for their ability to reverse or delay methylation-based biological aging. Clinical trials integrating methylation clocks as endpoints are beginning to evaluate the efficacy of these interventions, potentially enabling real-time monitoring of biological age and intervention impact. These efforts highlight the potential for DNA methylation biomarkers to serve as both diagnostic tools and therapeutic targets in aging medicine (Saliev and Singh, 2025).

As DNA methylation clocks move closer to clinical application, ethical and clinical implications must be carefully considered. It is possible that application will be regulated by ethical and moral norms or law in different countries. The use of biological age estimates raises questions about privacy, data security, and potential discrimination in insurance or employment based on aging biomarkers. Informed consent, equitable access, and the psychological impact of revealing accelerated biological aging to individuals are critical issues requiring clear guidelines and policies. It is possible that different countries may introduce laws or regulations to govern these studies and their applications. Furthermore, integrating methylation age into clinical decision-making necessitates robust validation and standardization to avoid premature or inappropriate use. Therefore, it should be considered only as an indicative or reference metric in age prediction.

Looking ahead, the prospects for advancing aging research and medicine through DNA methylation are profound. Improved clock models incorporating multi-omics data and diverse populations will enhance accuracy and inclusivity (Chen et al., 2023). While early epigenetic clocks may improve in accuracy over time, CpG sites with higher variability can still be important for capturing disease-related changes. In fact, some variable CpGs may reflect environmental exposures, inflammation, or other pathological processes, making them valuable for linking biological age to chronic disease risk. However, their contribution to age prediction may be down-weighted in refined clocks focused on stable age-associated CpGs.

Large-scale, multi-population validation to ensure accuracy across ethnicities and age ranges. Personalized aging metrics will enable early detection of disease risk and guide targeted prevention, ultimately extending health span and reducing the burden of age-related diseases. For example, hundreds of blood CpG sites are associated with Alzheimer’s disease risk (Sun et al., 2023); differential methylation at CpG sites is associated with chronic conditions such as diabetic kidney disease, where specific CpGs predict progression or risk beyond age effects (Smyth et al., 2022). It is necessary to standardize protocols for sample collection, processing, and analysis. Additionally, understanding the mechanistic underpinnings of methylation changes may reveal new therapeutic targets to modify the aging process itself (McGee et al., 2024). Integration with electronic health records and decision-support systems will benefit clinic application. Collaborative efforts spanning molecular biology, epidemiology, clinical research, and bioethics will be essential to realize these goals.
